# A fusion protein composed of the DSL domain of Dll1 and RGD motif protects cryptic stem cells in irradiation injury

**DOI:** 10.1042/BSR20171255

**Published:** 2018-03-09

**Authors:** Zhi-Jian Sun, Yi-Zhe Zhang, Fan Liu, Juan-Juan Chen, Dong-Xue Chen, Hong-Bao Liu, Liang Liang, Hua Han

**Affiliations:** 1 State Key Laboratory of Cancer Biology, Department of Medical Genetics and Developmental Biology, Fourth Military Medical University, Xi’an 710032, China; 2 Department of Research Center for Reproductive Medicine, No. 202 Hospital of PLA, Shenyang 110003, China; 3 Department of Nephrology, Xijing Hospital, Fourth Military Medical University, Xi’an 710032, China

**Keywords:** cryptic stem cells, DSL domain, irradiation, Notch signal, RGD motif

## Abstract

Intestine is vulnerable to irradiation injury, which induces cell death and compromises regeneration of intestinal crypts. It is well accepted that cryptic stem cells, which are responsible for cryptic regeneration under physiological and pathological conditions, are controlled by multiple cell-intrinsic and environmental signals such as Notch signaling. Therefore, in the present study, we tested whether a soluble Notch ligand tethered to endothelial cells—mD1R—the Delta–Serrate–Lag2 (DSL) domain of mouse Notch ligand Delta-like1 fused with a RGD motif could protect cryptic cells from irradiation-induced intestinal injury. The result showed that administration of mD1R, which activated Notch signaling in intestinal cells, ameliorated loss of body weight and reduction of cryptic structures in intestine after total body irradiation (TBI) in mice. Histological staining showed that injection of mD1R after TBI promoted cryptic cell proliferation and reduced cell apoptosis in crypts. Immunofluorescence staining and reverse transcription (RT)-PCR showed that mD1R increased the level of Lgr5, Bmi1, Olfactomedin-4 (OLFM4), and IRIG1 in crypts, suggesting a protective effect on cryptic stem and progenitor cells after irradiation. Moreover, we found that administration of mD1R increased the number of Paneth cells and the mRNA level of Defa1, and the number Alcian Blue+ Goblet cells decreased first and then increased after irradiation, suggesting that mD1R promoted the maturation of the intestinal crypt after irradiation injury. Our data suggested that mD1R could serve as a therapeutic agent for the treatment of irradiation-induced intestinal injury.

## Introduction

The intestinal epithelium covering the intestinal lumen is essential for the absorption of nutrients. Due to continuous exposure to toxic substances in foods, this monolayer of epithelial cells undergo quick replenishment with nearly 90% of the intestinal epithelium shedding off every 3–4 days, which is replaced by cells newly generated from the crypt epithelium. It is believed that cell consumption of every villus is supported by at least six crypts. In addition, exposure to high-dose ionizing radiation (IR) can cause lethal GI injury, a process known as radiation GI syndrome. Crypts shrink progressively during the first 24–48 h after irradiation. DNA repair-induced apoptosis of mitotic cells in crypts is generic after irradiation, resulting in failure of epithelial regeneration and even death. The molecular mechanism for IR-induced intestinal injury has not been well understood, and the clinical treatment has been unsatisfactory so far.

Under physiological conditions, intestinal stem cells (ISCs) harbored in the crypt bottom are responsible for the replenishment of intestinal epithelial cells. Several types of stem and progenitor cells have been identified in the intestinal crypts. The crypt base columnar (CBC) stem cells or regular stem cells are often recognized as Lgr5 (Leu-rich repeat containing G protein-coupled receptor 5)-positive, and are intermingled with Paneth cells at the base of the crypt. These cells also express the leucine rich repeats and immunoglobulin-like domains 1 (LRIG1), a single-pass transmembrane receptor, and Olfactomedin-4 (OLFM4). The Lgr5+ CBC cells can generate all epithelial lineages over a 60-day period, suggesting that they represent the stem cell population of the intestine. Lgr5+ CBC cells generate rapidly cycling progenitor cells or transit-amplifying (TA) cells, which undergo approximately 4–5 rounds of rapid cell division while migrate up the crypt–villus axis. After crossing the crypt–villus boundary, these cells terminally differentiate into different mature epithelial cell populations, including absorptive enterocytes, Goblet cells, enteroendocrine cells, and Paneth cells [1,2]. Another population of stem cells in the crypt has been known as TA +4 stem cells expressing Bmi1, which occupy a position of the fourth from the crypt base. This Bmi1+ population is relatively quiescent, radiation resistant, and not regulated by Wnt signaling. It is believed that +4 cells could be activated under tissue injury to rapidly regenerate the Lgr5+ CBC stem cell pool and restore epithelial renewal [1,3]. They are therefore considered as a reserving stem cell population that is different from the Lgr5+ CBC compartment at the crypt base.

Like other adult stem cells, ISCs are under strict control by cell-intrinsic programs and intercellular signaling. For instance, p53, PUMA, PI3K/Akt, β-arrestin 2, as well as CDK4/6 have been shown to participate in the radiosensitivity of ISCs. In addition, signaling molecules mediating cell–cell communications, such as Wnts, BMP, Noggin, and Eph-Ephrins, have also been implicated in the regulation of ISCs [4]. The Notch pathway is also important in regulating proliferation as well as lineage commitment within the intestine. The Notch signaling pathway is highly conserved and regulates stem cell fates through lateral inhibition [5]. In mammals, four Notch receptors (Notch1–4) and five ligands (Jagged1, 2, and Delta-like 1, 3, and 4) have been identified. Notch1 and Notch2 at a weaker level, is expressed in crypt epithelial cells. The Notch ligands Dll 1 and 3, and Jag1 are also expressed in crypt epithelium [6,7], and Notch signaling is active in ISCs as well as crypt progenitors [8–10]. Moreover, Dll1 and Dll4 are expressed in intestinal secretory cells, including Paneth cells and crypt base goblet cells that act as niche cells to support active stem cells [2,11]. Conditional knockout of the transcription factor recombination signal binding protein Jκ (RBP-J) results in secretory cell expansion at the expense of enterocytes and epithelial proliferation [12]. Simultaneous deletion of Notch1 and Notch2, Dll1 and Dll4 deletion, or treatment with γ-secretase inhibitor (GSI) exhibits similar phenotypes [13–15]. Conversely, mutations causing constitutive Notch activation result in depletion of secretory lineage cells and increased proliferation [16,17]. Interestingly, when Notch activation was inhibited, cryptic cells proliferation decreased dramatically, and CBCs were extinguished [9,12,14].

The recognition of Notch receptors is mediated by a conserved Delta–Serrate–Lag2 (DSL) domain located on the N terminal of the Notch receptor. Canonical Notch activation involves consecutive enzymatic receptor cleavages within the transmembrane domain executed by γ-secretase-mediated reactions. This process requires endocytosis of the Ligand–Notch extracellular domain in the signal-sending cell, and releases Notch intracellular domain (NICD) that subsequently translocates into the nucleus of the signal-accepting cell. NICD then interacts with RBP-J and transactivates the target genes such as the hairy and enhancer of split (Hes) family basic helix–loop–helix (bHLH) factors [18]. To efficiently activate Notch receptors *in vivo*, we have developed a fusion protein, the mouse D1R (mD1R), which is composed of the DSL domain of mouse Dll1 and an arginine-glycine-aspartic acid (RGD) nonapeptide (CRGDCGVRY) motif recognizing the endothelial integrin αvβ3 and triggering ligand endocytosis [19,20]. We have demonstrated that mD1R promotes hematopoietic stem cells (HSC) expansion *ex vivo* and engraftment *in vivo* after transplantation [21]. In the present study, we tested whether this polypeptide could protect ISCs in irradiation injury.

## Materials and methods

### The production of recombinant mD1R

The production of mD1R has been described previously [21,22]. In brief, the mouse Dll1 DSL domain (amino acids 126–224) was amplified and fused with a RGD-coding fragment by PCR to obtain mD1R gene. To construct pET32a-mD1R, the mD1R gene fragment was cloned into the pET32a(+) between the Nco I and Xho I sites. *Escherichia coli* BL21(DE3) were then transformed with the plasmid and positive clones were cultured and induced with isopropyl β-D-thiogalactoside (IPTG) for the production of the mD1R protein. The protein was purified using Ni^2+^-NTA columns (Invitrogen, Carlsbad, CA) as a Trx-tagged protein according to the manufacturer’s manual and then cleaved by thrombin (Novagen, Darmstadt, Germany), and purified using Ni^2+^-NTA columns to obtain the S-tagged protein ultimately.

### Mouse irradiation

C57BL/6 mice were maintained under specific-pathogen-free (SPF) conditions. Male mice with age of 8 to 10 weeks were subjected to total body irradiation (TBI) with γ-radiation from a ^60^Co irradiator at a dosage of 8, 10, or 12 Gy. The mice were then maintained with water containing gentamycin sulfate (10μg/mL). The mice were injected intraperitoneally (i.p.) with mD1R (100 μg/mouse) [22] or phosphate-buffered saline (PBS) 2 h postirradiation, and this was followed by daily injection of the same reagent until the end of the experiment. All animal experiments were approved by and performed in accordance with guidelines from the Animal Experiment Administration Committee of the Fourth Military Medical University.

### Histology

Mice were perfused with 4% paraformaldehyde (PFA) and killed humanly. The intestine tissues were removed immediately and fixed further in Bouin’s fixative overnight. Samples were then embedded in paraffin and cut into 5 μm-thick section, then stained with hematoxylin and eosin (H&E) staining according to a standard protocol. For staining with Alcian Blue, sections were dipped in the Alcian Blue solution (pH 2.5) for 5 min, and then counterstained in 0.1% nuclear fast red for 5 min, followed by washing in water. Images were taken under a microscope with a CCD camera.

For immunofluorescence and immunohistochemistry staining, sections were washed and blocked for 2 h at room temperature. The sections were incubated with differentially conjugated antibodies for 2 h at room temperature. After washing, the slides were counterstained with Hoechst33258 and mounted. The antibodies included anti-Ki67-FITC (Millipore), anti-Caspase-3-PE (CST-9662S), anti-β-catenin-FITC (Millipore), anti-Lgr5-FITC (Abcam, ab75732), anti-BMI1-FITC (Abcam, ab14389), anti-OFLM4-FITC (NOVUS, NBP2-24535), anti-NICD (CST-3608), and anti-LRIG1-PE (RD-AF3688). For the staining of apoptotic cells in the intestinal tissue sections, a terminal deoxynucleotidyltransferase-mediated dUTP nick-end labeling (TUNEL) kit (Promega, Madison, WI) was employed according to the manufacturer’s protocol. Images were taken using a fluorescence microscope (Olympus BX51, Japan) with a CCD camera (Olympus DP70) or a confocal microscope (FV1000, Olympus).

### Real-time reverse transcription (RT)-PCR

Total RNA was prepared from the intestine tissues by using the Trizol reagent (Invitrogen, Carlsbad, CA) according to the manufacturer’s instructions. Complementary DNA was prepared using a reverse transcription kit (Takara, Otsu, Japan). Real-time PCR was performed by using a kit (SYBR Premix EX Taq, Takara) and the ABI PRISM 7500 real-time PCR system, with β-actin as a reference control. Primers used in real-time PCR included: β-actin, 5′-CATCCGTAAAGACCTCTATGCCAAC and 5′-ATGGAGCCACCGATCCACA; Hes1, 5′-TCCAGGATGAGGACATGAGCAC and 5′-GAACGTCACACACCAGCAGGTTA; LGR5, 5′-CAGGAGGGAGAACAGAAACTCCA and 5′-CCTGGTTGGCTGCTTGCTT; Defa1, 5′-CTGCCGGAGTCTGACTGGAA and 5′-ATCAGTCCCACTGTCTGTCTCAATG; BMI1 [23], 5′-CCAATGAAGACCGAGGAGAA and 5′-TTTCCGATCCAATCTGCTCT.

### Statistics

Statistical analysis was performed with the SPSS 12.0 program. Results were expressed as the means ± SD. To quantify histological images, at least five randomly selected fields of each section were counted under microscope. The comparisons between groups were undertaken using the unpaired Student’s *t* test. *P*<0.05 was considered statistically significant.

## Results

### mD1R attenuated irradiation-induced intestinal injury in mice

**Figure 1:**
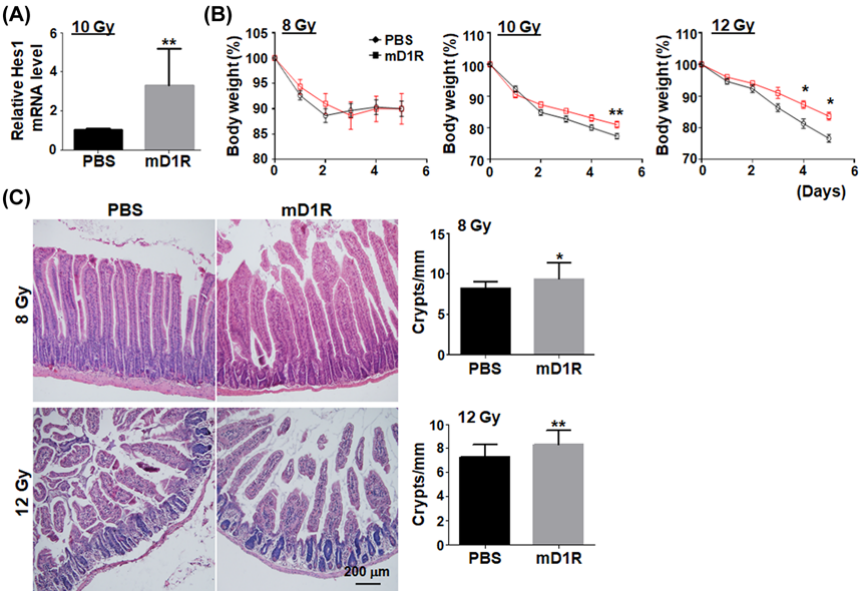
mD1R attenuated irradiation-induced intestinal injury in mice (**A**) Normal mice were irradiated by total body irradiation (TBI) with 10 Gy of γ-ray and injected i.p. with PBS or 100 μg of mD1R every day from the first day to the fifth day after the irradiation. The expression of Hes1 mRNA in intestine was determined by real time RT-PCR. (**B**) Mice were irradiated with 8, 10, or 12 Gy of γ-ray and injected i.p. with PBS or 100 μg of mD1R every day after the irradiation. The body weight of the mice was determined on the indicated days. (**C**) Mice were irradiated by TBI with 8 or 12 Gy of γ-ray and injected i.p. with PBS or 100 μg of mD1R every day from the first day after the irradiation. The intestine samples of the mice were collected on the fifth day after the irradiation, and subjected to H&E staining. The number of crypts in 1 mm length of intestine was counted and compared between the two groups; bars = mean ± SD (*n*=3); *P<0.05, **P<0.01.

mD1R is a polypeptide that is designed to activate Notch signaling in endothelial cells with relative specificity. No gross changes including body weight have been noticed in our previous studies [21,22] (data not shown). To examine whether mD1R could protect mice from irradiation-induced intestinal injury, normal mice were subjected to TBI of 10 Gy, and injected i.p. with mD1R (100 μg) or PBS every day to activate Notch signaling. On the fifth day after irradiation and mD1R injection, the expression of Hes1 mRNA was significantly up-regulated in intestinal cells (Figure 1A). Immunohistochemistry of cleaved NICD indicated that Notch signaling was activated in the intestinal epithelia, especially around the base of crypts (Supplementary Figure S1). This suggested that, although approximately, injection of mD1R could activate Notch signaling, consistent with the observation in other tissues such as bone marrow [21].

Next, mice were irradiated with different dosages (8, 10, and 12 Gy) of γ-ray and injected i.p. with mD1R (100 μg) or PBS every day. The body weight of the mice, which is a general indication of irradiation-induced intestinal injury, was determined on the indicated days after irradiation and mD1R administration. The result showed that the injection of mD1R protected the mice from irradiation-induced body weight loss after 10 and 12 Gy of γ-ray (Figure 1B). Irradiation with 8 Gy induced a mild body weight loss on day 2 but mD1R did not show significant effect at this dosage point (Figure 1B). Irradiation with 14 Gy induced animal death within 5 days after irradiation in the study, which could not be rescued by mD1R (data not shown). To further examine the effect of mD1R on irradiation-induced intestinal injury, we performed histological observation of the crypt and villus compartments of small intestine after irradiation with γ-ray. H&E staining showed that mice accepting mD1R had more intestinal crypts after irradiation, as compared with the irradiated mice accepting PBS (Figure 1C). These data suggested that mD1R, which likely activated Notch signaling in intestinal crypts, could reduce the radiation-induced crypt damage within certain IR dose range.

### mD1R enhanced proliferation and reduced apoptosis of cells in intestinal crypts after irradiation in mice

**Figure 2:**
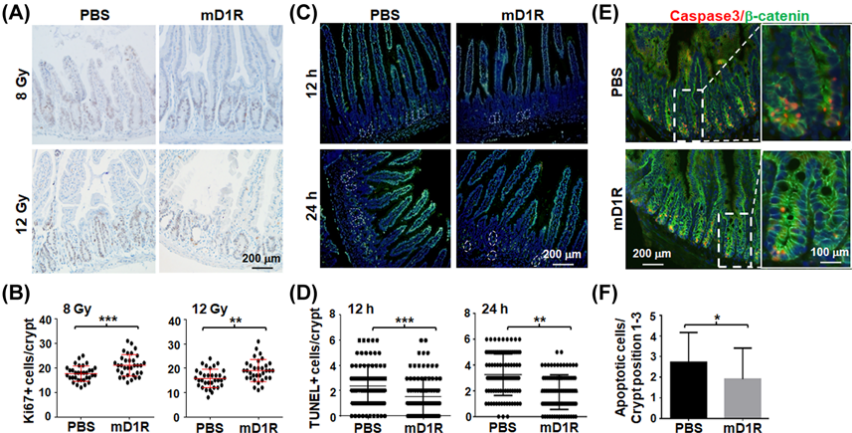
mD1R enhanced the proliferation and reduced the apoptosis of intestinal cryptic cells after irradiation in mice (**A** and **B**) Mice were irradiated by TBI with 8 or 12 Gy of γ-ray and injected daily i.p. with PBS or 100 μg of mD1R from the first day after the irradiation. On the third day after irradiation, the intestine of the mice were collected, and stained by immunohistochemistry with anti-Ki67 (A). The number of Ki67+ cells per crypt was counted and compared between the two groups (B). (**C** and **D**) Mice were irradiated by TBI with 10 Gy of γ-ray and injected i.p. with PBS or 100 μg of mD1R immediately after the irradiation. The intestine samples of the mice were collected on 12 and 24 h after the irradiation and stained with TUNEL. Nuclei were counter-stained with Hoechst33258 (C). The number of TUNEL+ cells per crypt was counted and compared between the two groups (D). (**E** and **F**) Samples in (C) were stained by immunofluorescence with anti-Caspase 3 and anti-β-catenin. Nuclei were counter-stained with Hoechst33258 (E). The number of apoptotic cells per crypt was counted and compared (F); bars = mean ± SD (*n*=5); *P<0.05, **P<0.01, ***P<0.001.

Ionizing radiation inhibits cell proliferation and induces apoptosis through p53. We therefore examined the effects of mD1R on cryptic cell proliferation and apoptosis. Mice were subjected to TBI with 8 or 10 Gy γ-ray, and cell proliferation in intestinal crypts was determined by immunohistochemistry using the anti-Ki67 antibody on day 3 after the irradiation. The result showed that administration of mD1R increased the number of Ki67+ cells in intestinal crypts (Figure 2A,B), suggesting that mD1R promoted cryptic cell proliferation after irradiation.

Next we determined apoptosis of cryptic cell after TBI by TUNEL staining. Mice were irradiated by TBI with 10 Gy γ-ray and injected i.p. with mD1R or PBS immediately. Intestinal tissues were collected at 12 and 24 h after the irradiation, and subjected to the TUNEL assay. The result showed that injection of mD1R significantly reduced TUNEL+ cells in intestinal crypts as compared with mice injected with PBS (Figure 2C,D), suggesting attenuated apoptosis. This was supported by immunofluorescence staining of Caspase-3, a critical proteinase executing apoptosis. As shown in Figure 2E,F, administration of mD1R significantly reduced Caspase-3+ cells in β-catenin+ cryptic cell compartment (position 1–3). Collectively, these data suggested that administration of mD1R could ameliorate irradiation-induced intestinal injury by promoting cell proliferation and reducing cell apoptosis in intestinal crypts in mice.

### mD1R protected cryptic stem cells during irradiation damage

**Figure 3:**
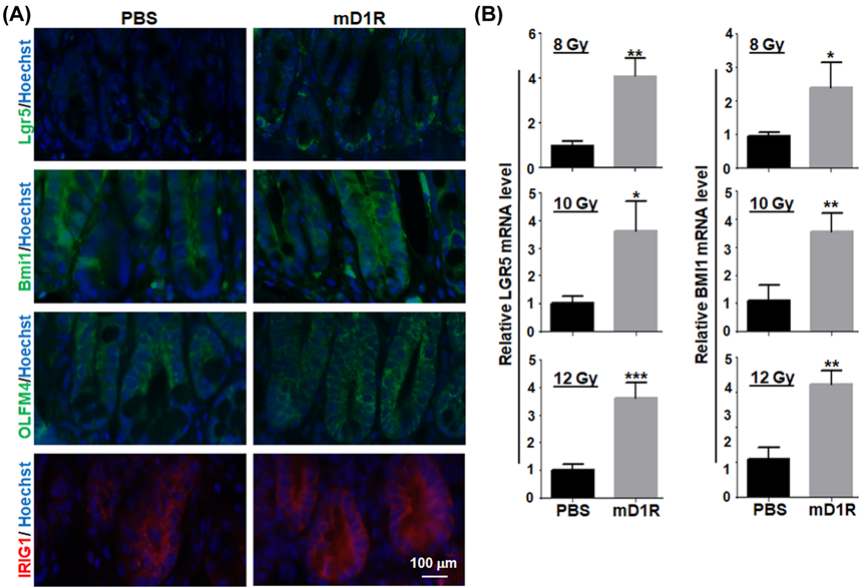
mD1R protected cryptic stem cells after irradiation (**A**) Normal mice were irradiated by TBI with 8, 10, or 12 Gy of γ-ray and injected i.p. with PBS or 100 μg of mD1R every day from the first day after the irradiation. On the third day post irradiation, the intestine samples of the mice were collected, and stained by immunofluorescence with anti-Lgr5, anti-Bmi1, anti-OLFM4, or anti-IRIG1. Pictures show the morphology of intestine from the 12 Gy group. (**B**) Total RNA was prepared from the intestine tissue from mice in (A). The expression of Lgr5 and Bmi1 mRNA in intestine was determined by using real time RT-PCR; bars = mean ± SD (*n*=5); *P<0.05, **P<0.01, ***P<0.001.

The homeostasis and regeneration of intestinal mucosa depend on a group of cryptic stem and progenitor cells that are regulated by cell-intrinsic programs and microenvironment. To examine whether the endothelia-targeted Notch ligand could influence cryptic stem cells, mice were irradiated with 12 Gy of γ-ray and treated with mD1R by daily injection i.p. for 3 days. Immunofluorescence staining using antibodies recognizing specific markers on cryptic stem and progenitor cells showed that a significantly stronger Lgr5-positive signals and Bmi1-positive signals were detected within the intestinal crypts of irradiated mice injected with mD1R than with PBS. The levels of OLFMA4 and IRIG1 were also higher in the mice injected with mD1R than with PBS (Figure 3A). Then, after different dosages (8, 10, and 12 Gy) of γ-ray irradiation and mD1R injection, the intestinal cryptic cells were isolated and subjected to qRT-PCR analysis. The result showed that the relative mRNA levels of Lgr5 and Bmi1 in cryptic cells were significantly higher in mice injected with mD1R than with PBS (Figure 3B). These data suggested that mD1R protected the intestinal cryptic stem cells after irradiation injury.

### mD1R improved crypt maturation after irradiation

**Figure 4:**
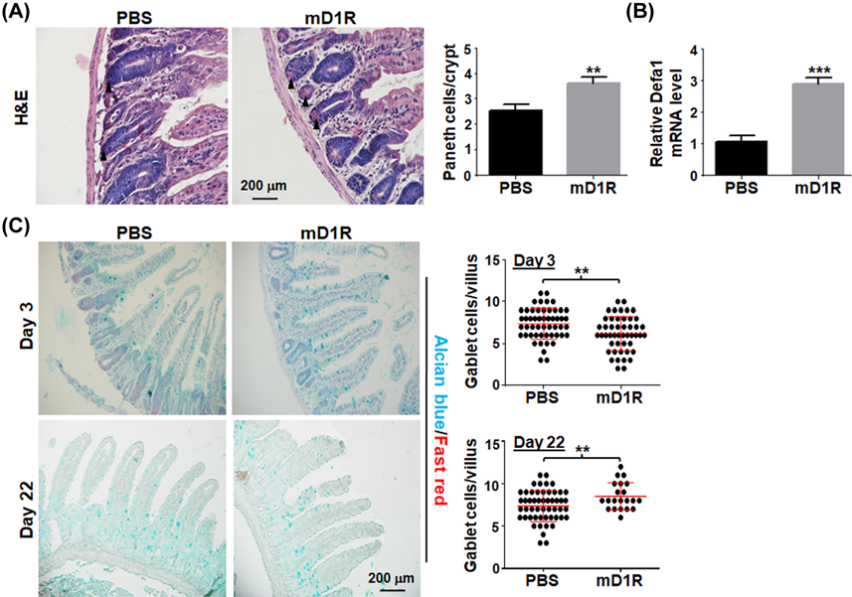
mD1R improved crypt maturation after irradiation (**A**) Normal mice were irradiated by TBI with 12 Gy of γ-ray and injected i.p. with PBS or 100 μg of mD1R every day from the first day after the irradiation. Three days post the irradiation, the intestine of the mice was collected and subjected to H&E staining. The Paneth cells were indicated with arrowheads, and the number of Paneth cells was counted and compared between the two groups. (**B**) Total RNA was prepared from the intestine tissue in (A). The expression of Defa1 mRNA in intestine was determined by using real time RT-PCR. (**C**) The intestine was collected from mice in (A) on day 3 and 22 after the irradiation. The tissue sections were stained with Alcian Blue/Fast red. The number of Goblet cells per villus was compared; bars = mean ± SD (*n*=5); **P<0.01, ***P<0.001.

We then determined whether mD1R could promote the regeneration of intestinal crypt. Normal mice were irradiated by TBI with 12 Gy of γ-ray and injected i.p. with mD1R or PBS every day for 3 days. With H&E staining, the mice intestine were collected and stained. The result showed that Paneth cells increased significantly in the intestinal cryptin mD1R-treated mice as compared with the PBS-treated group (Figure 4A), suggesting increased regeneration of Paneth cells after mD1R treatment. This was further supported by qRT-PCR assay of the Paneth cell marker *Defa1*, which showed that mD1R treatment increased the expression of *Defa1* (Figure 4B). Moreover, we examined the number of Goblet cells by Alcian Blue/Fast red staining. The result showed that treatment with mD1R increased the number of Alcian Blue+ Goblet cells per villus 22 days after the irradiation, although the Goblet cell number decreased at day 3 after irradiation (Figure 4C). These results suggested that mD1R increased crypt regeneration and maturation, likely through protection of cryptic stem cells after irradiation injury.

## Discussion

The intestinal epithelium undergoes constant and rapid turnover that is supported by ISCs accommodated in the intestinal crypts. Loss of cryptic stem and progenitor cells results in disruption of the intestinal epithelium, a primary radiotherapy-associated pathogenesis in clinical use of radiotherapy for treating abdominal and pelvic cancers [24]. Although the underlying molecular mechanism of IR-induced intestinal injury is still not completely clear, it is believed that Lgr5+ especially the +4 ISCs are relatively quiescent and are not highly sensitive to irradiation injury, but their offspring TA progenitors cells are highly proliferative and therefore sensitive to irradiation [25]. Notch signaling, in collaboration with other pathways such as Wnt and BMP signaling pathways, plays multiple roles in the regulation of proliferation and differentiation of cryptic stem and progenitor cells [26]. Therefore, in the present study, we tested the role of an EC-tethered soluble Notch ligand in irradiation-induced intestinal injury. Our data have shown that injection of mD1R displayed a protective role of mice in irradiation-induced injury. mD1R could trigger Notch signaling in cryptic stem and progenitor cells, and protect cryptic stem and progenitor cells by promoting cell proliferation and the maturation of crypts after irradiation.

It has been generally supposed that ISCs and cryptic progenitor cells are vulnerable to IR insult [27]. However, evidence also suggests that IR targets initially the vascular endothelial cells in the crypt–villus axis [28]. The essential role of endothelial cells that line capillaries is serving as conduits for bloodstream. However, tissue-specific endothelial cells also produce different types of growth factors and other secreted molecules, known as angiocrine factors, to regulate surrounding stem cells and tissue parenchymal cells. During injury induced by various insults, these EC-derived factors modulate the self-renewal and differentiation of tissue stem and progenitor cells, and participate in the induction, specification, patterning, and guidance of organ regeneration [29]. Inside the villus endothelial cells produce ceramide catalyzed by acid sphingomyelinase (ASMase), which is suggested to initiate GI syndrome. Treatments that reduce ceramide, for instance, genetic ASMase deficiency and administration of anticeramide antibody, ameliorate IR-induced endothelial apoptosis and protect intestine in IR-induced damage [30]. Notch signaling has long been recognized as a critical regulator of endothelial cells in both vessel morphogenesis and homeostasis. Recent evidence has also shown that Notch signaling regulates angiocrine function of endothelial cells [22]. It is therefore possible that mD1R protect cryptic stem and progenitor cells during irradiation-induced injury by modulating the production of paracrine factors derived from endothelial cells. The proregeneration effect of mD1R would be indirect, and therefore could be weak. This could also explain the phenomenon that Notch activation was detected in the epithelial and cryptic cells after application of mD1R.

The DSL domain of Notch ligands is responsible for receptor binding and triggering. Recombinant soluble Notch ligands have been developed by using the Notch extracellular domain fused with the Fc fragment and the DSL domain. It has been shown that maximal Notch activation requires ligand endocytosis in addition to ligand–receptor binding. We have designed the new type of soluble Notch ligand that is composed of the DSL domain of Dll1 and a RGD motif to meet these two demands of Notch receptor activation. D1R has a stronger activity to activate Notch signaling and to enhance HSC expansion *ex vivo*, likely due to the dynamin-dependent endocytosis mediated by RGD-integrin αvβ3 interaction. Moreover, the RGD motif targets the D1R protein to ECs and provides EC-specificity of D1R. The D1R peptide can be conveniently manufactured in *E. coli* and purified, guaranteeing its medical application potential. We have recently shown that protective role of D1R in the irradiation-induced bone marrow injury. Here, we found that D1R is also protective in irradiation-induced intestinal injury. Therefore, D1R might be a candidate for clinical use in the therapy of irradiation injury in future. In contrast, as shown in our previous study, DSL of Notch1 without a RGD motif could not efficiently bind to ECs and failed to activate Notch signaling [22], therefore might not function in radioprotection as the D1R peptide. This is likely due to the facts that activation of Notch receptors requires ligand-mediated endocytosis [31, 32].

## Supporting information

**Supplementary Figure S1:** **Immunohistochemistry of NICD in intestine samples.** Mice were injected i.p with mD1R (mg/Kg) 3 days post 12 Gy irradiation. Intestine samples were collected and stained with anti-NICD immunohistochemistry. Positive signals are indicated with white arrows.

## Abbreviations

CBC: crypt base columnar
DSL: Delta–Serrate–Lag2
GSI: γ-secretase inhibitor
H&E: hematoxylin and eosin
Hes: hairy and enhancer of split
HSC: hematopoietic stem cell
IR: ionizing radiation
ISC: intestinal stem cell
LRIG1: leucine rich repeats and immunoglobulin-like domains 1
NICD: Notch intracellular domain
OLFM4: Olfactomedin-4
PBS: phosphate-buffered saline
RBP-J: recombination signal binding protein Jκ
RGD: arginine-glycine-aspartic acid
TA: transit-amplifying
TBI: total body irradiation
