# Supplementary material for: A fusion protein composed of the DSL domain of Dll1 and RGD motif protects cryptic stem cells in irradiation injury
Source: Biosci Rep. 2018 Mar 9;38(2):BSR20171255. doi: 10.1042/BSR20171255 (PMC5843746; doi:10.1042/BSR20171255)
Supplement: Supplementary file 1 [file bsr20171255_Supp1.pdf]

Supplementary materials

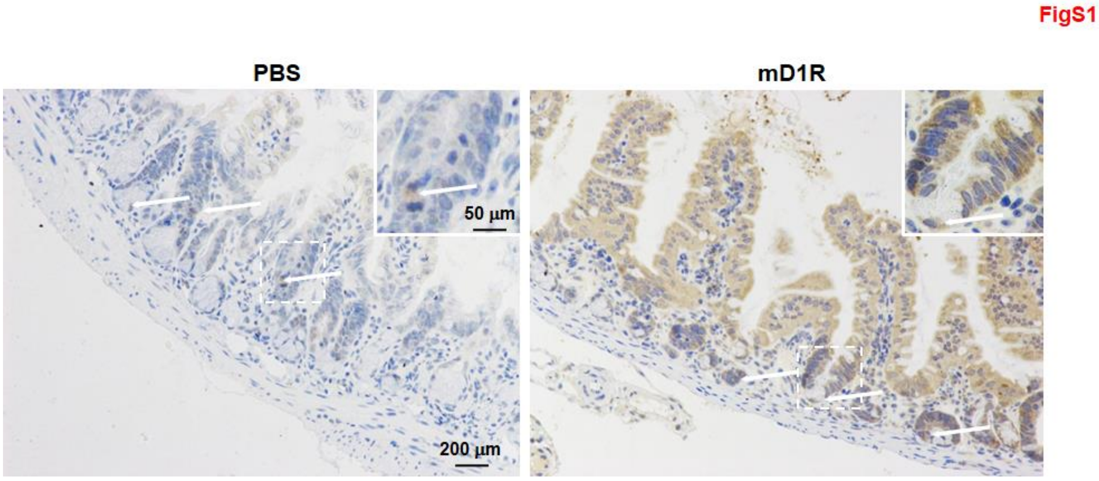

Supplementary Figure S1: Immunohistochemistry of NICD in intestine samples. Mice were injected i.p with mD1R (mg/Kg) 3 days post 12 Gy irradiation. Intestine samples were collected and stained with anti-NICD immunohistochemistry. Positive signals are indicated with white arrows.
